# The Montreal Cognitive Assessment (MoCA) - A Sensitive Screening Instrument for Detecting Cognitive Impairment in Chronic Hemodialysis Patients

**DOI:** 10.1371/journal.pone.0106700

**Published:** 2014-10-27

**Authors:** Frances E. Tiffin-Richards, Ana S. Costa, Bernhard Holschbach, Rolf D. Frank, Athina Vassiliadou, Thilo Krüger, Karl Kuckuck, Theresa Gross, Frank Eitner, Jürgen Floege, Jörg B. Schulz, Kathrin Reetz

**Affiliations:** 1 Department of Neurology, RWTH Aachen University Hospital, Aachen, Germany; 2 Jülich Aachen Research Alliance (JARA) – Translational Brain Medicine, Aachen and Jülich, Germany; 3 KfH Curatorship for Dialysis and Kidney Transplant e.V., KfH-Nephrology Center, Stolberg, Germany; 4 Department of Internal Medicine, St.-Antonius-Hospital Eschweiler, Eschweiler, Germany; 5 Dialysis Center Aachen, Dialysis Center, Aachen, Germany; 6 Division of Nephrology and Clinical Immunology, RWTH Aachen University, Aachen, Germany; 7 Department of Internal Medicine, Dresden-Friedreichstadt Hospital, Dresden, Germany; 8 Bayer Pharma AG, Global Drug Development, Kidney Diseases Research, Wuppertal, Germany; 9 Institute of Neuroscience and Medicine (INM-4), Research Center Jülich GmbH, Jülich, Germany; University of Manchester, United Kingdom

## Abstract

**Background:**

Chronic kidney disease (CKD) patients undergoing hemodialysis (HD) therapy have an increased risk of developing cognitive impairment and dementia, which are known relevant factors in disease prognosis and therapeutic success, but still lack adequate screening in clinical routine. We evaluated the Montreal Cognitive Assessment (MoCA) for suitability in assessing cognitive performance in HD patients in comparison to the commonly used Mini-Mental State Examination (MMSE) and a detailed neuropsychological test battery, used as gold standard.

**Methods:**

43 HD patients and 42 healthy controls with an average age of 58 years, were assessed with the MoCA, the MMSE and a detailed neuropsychological test battery, covering the domains of memory, attention, language, visuospatial and executive functions. Composite scores were created for comparison of cognitive domains and test results were analyzed using Spearman's correlation and linear regression. Cognitive dysfunction was defined using z-score values and predictive values were calculated. Sensitivity and specificity of the MoCA were determined using receiver operating characteristic (ROC) analysis.

**Results:**

HD patients performed worse in all cognitive domains, especially in memory recall and executive functions. The MoCA correlated well with the detailed test battery and identified patients with cognitive impairment with a sensitivity of 76.7% and specificity of 78.6% for a cut-off value of ≤24 out of 30 points. In the detailed assessment executive functions accounted significantly for performance in the MoCA. The MMSE only discriminated weakly between groups.

**Conclusions:**

The MoCA represents a suitable cognitive screening tool for hemodialysis patients, demonstrating good sensitivity and specificity levels, and covering executive functions, which appear to play an important role in cognitive performance of HD patients.

## Introduction

The association of cognitive impairment and a higher incidence of dementia in patients with chronic kidney disease (CKD) has been increasingly acknowledged over the last few years [1]–[3] and represents an important issue in an already vulnerable population. The prevalence of cognitive impairment in chronic hemodialysis (HD) patients has been estimated at 30–80% [4]–[7]. In addition to being associated with cerebrovascular disease and potentially other types of brain injury [8], cognitive impairment may jeopardize treatment adherence by affecting the efficiency of every-day tasks, including correct medication and dietary rules [9]. Moreover, cognitive impairment is a significant predictor of mortality in HD patients [5].

The call for early detection of cognitive impairment in patients with CKD has yet to be translated to every-day clinical practice. The necessity has, however, been voiced in earlier studies and the use of short and easy-to-apply cognitive screening tools has been suggested [5], [10]. The Montreal Cognitive Assessment (MoCA) [11] is a screening test for cognitive impairment that covers major cognitive domains including episodic memory, language, attention, orientation, visuospatial ability and executive functions, while remaining brief and easy to administer. It is generally considered superior to the well-established Mini-Mental State Examination (MMSE) screening test [12], [13], since the MoCA not only assesses executive functioning, which may be particularly important in the CKD population [14], [15], but also presents a higher sensitivity for mild cognitive impairment. Accordingly, the MoCA has been evaluated and found to be an adequate screening tool in various clinical populations, e.g. Alzheimer's dementia (AD) [16], cerebral small vessel disease [17], and other medical conditions such as cardiovascular disease [18], as well as being able to discriminate between mild cognitive impairment and elderly controls [19]. Recently, the MoCA was also recommended as a standardized approach to cognitive assessment in patients undergoing HD [20]. Therefore our primary goal was to further evaluate the MoCA as a brief screening tool for cognitive impairment in HD patients in comparison to a comprehensive cognitive testing. To achieve this, the ability to distinguish between HD patients with and without cognitive impairment, the sensitivity, specificity and predictive values of the MoCA were assessed. Additionally, psychometric criteria such as concurrent and criterion validity of performance on the MoCA, a comprehensive neuropsychological test battery and the standard brief cognitive screening test MMSE were evaluated.

## Subjects and Methods

### Ethics Statement

The research project was carried out in accordance with the latest version of the Declaration of Helsinki and approved by the ethics committee of the Medical Faculty of the RWTH Aachen University in Germany (EK 179/11). All participants gave informed, written consent before participating.

### Study population

Between February 2012 and March 2013, forty-eight patients on hemodialysis treatment were recruited from the Division of Nephrology of the RWTH Aachen University Hospital in Germany and three community based dialysis centers in the Aachen region. Forty-two matching healthy controls of varied intellectual and educational level, but without specific experience in neuropsychology, were recruited from the community and staff of the RWTH Aachen University. All participants with a history of neurological or psychiatric disease were excluded. In two cases of severe visual or motor impairment, specific tasks were not administered and therefore considered missing values. These cases included one patient with residual eye sight of 30% due to diabetic retinopathy and glaucoma and one patient with a disabilitating hand tremor.

### Clinical and demographic data

Medical history and demographic data were gained *via* self-report and/or from medical records. Patients' clinical data included medical history, current medication, CKD etiology, dialysis vintage, serum values of sodium, potassium, hemoglobin, hematocrit, creatinine, pH value and blood sugar and were obtained from routine clinical blood samples taken at the beginning of each dialysis treatment. The duration of dialysis treatment, ultrafiltration volume, pre-dialysis blood pressure values and intradialytic hypotensive episodes, defined as systolic blood pressure < 90 mmHg and/or diastolic blood pressure <50 mmHg, were collected from the dialysis protocols. Cardiovascular risk factors, including nicotine consumption, body mass index (BMI), serum cholesterol and high blood pressure were rated using the SCORE risk charts of the European Society of Cardiology [21] using the low risk chart for the German population. Hypertension was defined as the prescription of antihypertensive medication. Comorbidities were quantified using the Charlson Comorbidity Index (CCI) [22] corrected for dialysis [23], or age in the healthy control group [22].

### Neuropsychological testing

The neuropsychological test battery was administered to all subjects on a dialysis-free day by a psychologist or trained assistants. It consisted of two cognitive screening tests, the MoCA and the MMSE, as well as a detailed cognitive test battery that evaluated memory, language, attention, visuospatial ability and executive functions. As participants were partaking in a cross-sectional observational study with a repeated-measures design [24], previously validated alternate versions of the MoCA [25], as well as from other tests, were used to avoid practice effects. For all patients and controls the same order of test administration was used. The alternate versions of tests were contra-balanced in a pseudo-randomized order. Although all patients and controls underwent two rounds of testing, only the data of the first assessment was used in the current analyses and therefore not all participants completed the exact same version of all tests. Testing was performed in a quiet room with a low distraction level, but in cases of reduced mobility, testing was also performed in patients' hospital rooms. For the order of test administration, view List S1 in File S1.

The MoCA [11] is a brief screening tool assessing visuospatial and executive functions, attention, short-term memory, language and orientation, has been translated and adapted into several languages and is available freely on the Internet (http://www.mocatest.org). It includes tasks such as trail making test – part B, cube copying, clock drawing, naming, digit span backwards and forwards, serial subtraction, selective attention, sentence repetition, phonemic word fluency, verbal abstraction, a 5-word learning and delayed recall task, and spatial and temporal orientation. Completion time is approximately 10 to 15 minutes and a maximum of 30 points can be obtained.

The ability of the MoCA to screen for cognitive impairment in HD patients was to be evaluated through the comparison to a well-known screening test, the MMSE, and a detailed neuropsychological test battery. Given that our emphasis lay on the evaluation of the MoCA, detailed group analyses, such as correlation analysis, were not performed with the MMSE and the detailed neuropsychological battery.

The MMSE is a ten-minute screening test including questions to spatial and temporal orientation, immediate and delayed recall, language ability and oral command comprehension, serial subtraction and tasks to visuospatial ability. Here the German adaptation was used for all participants [12].

A more detailed analysis of the cognitive domains and psychometric characteristics, such as sensitivity, specificity and concurrent validity, of the MoCA was achieved by comparison to a detailed neuropsychological test battery. In the detailed neuropsychological test battery, verbal memory was examined using the California Verbal Learning Test (CVLT) [26], a word list recall task, and non-verbal memory was assessed with the Medical College of Georgia Complex Figures (MCGCF) test [27]. Attention was evaluated through a computer-based cued and non-cued reaction time (phasic and intrinsic alertness) task [28] and digit span forwards. Processing speed and executive functions were assessed using the Trail Making Task (TMT) forms A and B [29], phonemic and semantic word fluency [30], digit span backwards [31], and the interference task of the Stroop test [32]. The Boston Naming Test [33] gave information on overall language processing ability. Visuospatial abilities were tested with the copying task of the MCGCF and the Incomplete Letters subtest of the Visual Object and Space Perception (VOSP) test [34], which also served to identify individuals with visual impediments that could hinder further testing. We also included the Epworth Sleepiness Scale (ESS) [35] to measure daytime sleepiness. Subjective fatigue level was evaluated through a 10-point scale (0 = no fatigue to 10 = worst imaginable fatigue) adapted from the Brief Fatigue Inventory [36]. Depression and anxiety were quantified with the Hospital Anxiety and Depression Scale (HADS) [37]. For further detail on individual tests, see Table S1 in File S1.

### Statistical analysis

Data analysis was performed using Microsoft Excel 2010, IBM SPSS Statistics 20 for Windows and MedCalc Software Version 12.5.0. The distribution of the collected data was determined using the Kolmogorov-Smirnov test. Due to heterogeneous normal and non-normal distribution of data, non-parametric tests were used and all test results are presented with median and interquartile ranges for better comparability. Demographic data is presented according to the distribution of the respective data. Differences, and respective effect sizes (*r*), between patients and controls regarding demographic and clinical data, as well as the neuropsychological test scores, were computed using the non-parametric Mann-Whitney-U test with Bonferroni correction for multiple testing. The interpretation of effect sizes followed Cohen's proposal for *r* as small at 0.10–0.29, medium at 0.30–0.49 and large at >0.50 [38]. Correlation analyses were carried out using Spearman's rank correlation coefficient rho (*r_s_*). We calculated standard z-scores for each cognitive test, using the healthy control group as the reference group and without replacing missing values. Based on the median of z-scores of the individual tests, we calculated composite scores for each cognitive domain – memory, attention, language, visuospatial, executive functions – as well as two overall composite scores. The overall composite scores were an executive composite score, including the executive functions and attention tasks, and a non-executive composite score comprising of the language, visuospatial and memory tasks. The same procedure was used to create composite scores of the cognitive domains of the MoCA. For further analysis, cognitive dysfunction was defined using z-score values. Mild cognitive impairment was classified as z-scores one to two standard deviation (SD) below the norm and severe cognitive impairment as more than two SD below the norm in ≥2 neuropsychological tests [5], [9]. To examine concurrent validity between the MoCA total scores and the test battery overall composite scores, a bivariate linear regression model with the MoCA as the dependent variable and the composite scores as the independent variables was calculated. The raw total scores of the MoCA were used without education correction [16], and age, years of education, depression and fatigue levels were added as covariates [2]. To establish the discriminative validity of the MoCA in identifying patients with and without cognitive impairment, sensitivity and specificity levels were evaluated using the receiver operating curve (ROC) analysis and the criteria for cognitive impairment as described above. The cut-off score was determined based on maximal sensitivity and specificity. Positive and Negative Predictive Values (PPV/NPV) were calculated for the optimal cut-off value identified by the ROC analysis.

## Results

### Characterization of the HD patient sample

Forty-three HD patients completed testing and were included in the current analyses. Details of clinical and demographic data are presented in Table 1. In comparison to the control group, HD patients had a significantly higher rate of hypertension, diabetes type 2, hypercholesterolemia and nicotine consumption, yet no significant difference concerning BMI. Levels of fatigue and sleepiness, as well as scores on the depression scale were higher in the patient group. On average, patients had been on dialysis treatment for 50 months and spent four hours on dialysis, three times per week, with an average ultrafiltration volume of 1.2 liters. The 10-year risk of a fatal cardiovascular disease lay at 3–4% [21] while the CCI scoring system for HD patients presented a mean 10-year survival rate of 25% [23].

**Table 1:** Demographic and health characteristics.

Demographics	Patients	Controls	*p* e)
Age	58.3±13.9	57.9±11.8	.96
Gender	Male 52.1% (25)	Male 47.6% (20)	.67
Education (years)	12.0 (2.0)	13.0 (3.3)	.01
**Comorbidities**			
BMI	24.4 (6.7)	25.2±3.8	.71
Hypertension	83.3% (40)	33.3% (14)	<.001
Diabetes	45.8% (22)	4.8% (2)	<.001
Nicotine consumption	25.0% (12)	7.1% (3)	.02
Hypercholesterolemia	31.3% (15)	0%	<.001
Cardiovascular Score (ESC - SCORE)	1.0 (4.0)	-	-
Charlson Comorbidity Index (CCI)a)	4.0 (3.0)	-	-
**Hemodialysis**			
Dialysis Vintage (months)	36.0 (47.0)	-	-
Time on dialysis (hours)	4.0 (0.0)	-	-
Ultrafiltration volume (l)	1.2 (2.1)	-	-
**Etiology of renal disease**			
Diabetic nephropathy	27.1% (13)	-	-
Glomerulonephritis and systemic diseases	27.1% (13)	-	-
Vascular (hypertensive) kidney disease	14.6% (7)	-	-
Other causesb)	12.5% (6)	-	-
Polycystic kidney disease	10.4% (5)	-	-
Unknown	8.3% (4)	-	-
**Secondary diseases**			
Renal anemia	38.5% (20)	-	-
Secondary hyperparathyroidism	34.6% (18)	-	-
Renovascular hypertension	19.2% (10)	-	-
Kidney transplant in medical history	13.5% (7)	-	-
**Medication**			
Antihypertensive Medication (total number)	3.0 (4.0)	0.0 (0.0)	<.001
ACE* inhibitors	37.2% (16)	7.1% (3)	<.001
Beta blockers	67.4% (29)	16.7% (7)	<.001
Calcium channel blockers	48.8% (21)	7.1% (3)	<.001
Angiotensin receptor blockers	20.9% (9)	4.8% (2)	.03
Alpha-1 blockers	25.6% (11)	0%	<.001
Adrenergic alpha agonists	23.3% (10)	0%	<.001
Direct vasodilators	9.3% (4)	0%	.04
Diuretics	46.5% (20)	4.8% (2)	<.001
Antidiabeticsc)	37.2% (16)	2.4% (1)	<.001
Thyroid drugs	30.2% (13)	7.1% (3)	.01
Psychoactive drugsd)	23.3% (10)	0%	.02
Analgesics	16.3% (7)	2.4% (1)	.01
Glucocorticoids	25.6% (11)	0%	<.001

Notes. Values are presented as mean ± SD for normally distributed continuous variables, median (interquartile range) for non-normally distributed continuous variables and % (N) for percentages.

a) History of cerebral disease found in two cases;

b) Other causes include progression of acute kidney disease due to post-operative infections, urologic reflux diseases, analgesic medication;

c) Including oral antidiabetics and insuline therapie;

d) Psychoactive drugs include antipsychotics, antidepressants, anticonvulsives and drugs containing L-DOPA;

e) p-value of nonparametric Mann-Whitney-U test for independent samples;

* ACE = Angiotensin Converting Enzyme.

### Performance on the MoCA and association with demographic and clinical variables

**Figure 1:**
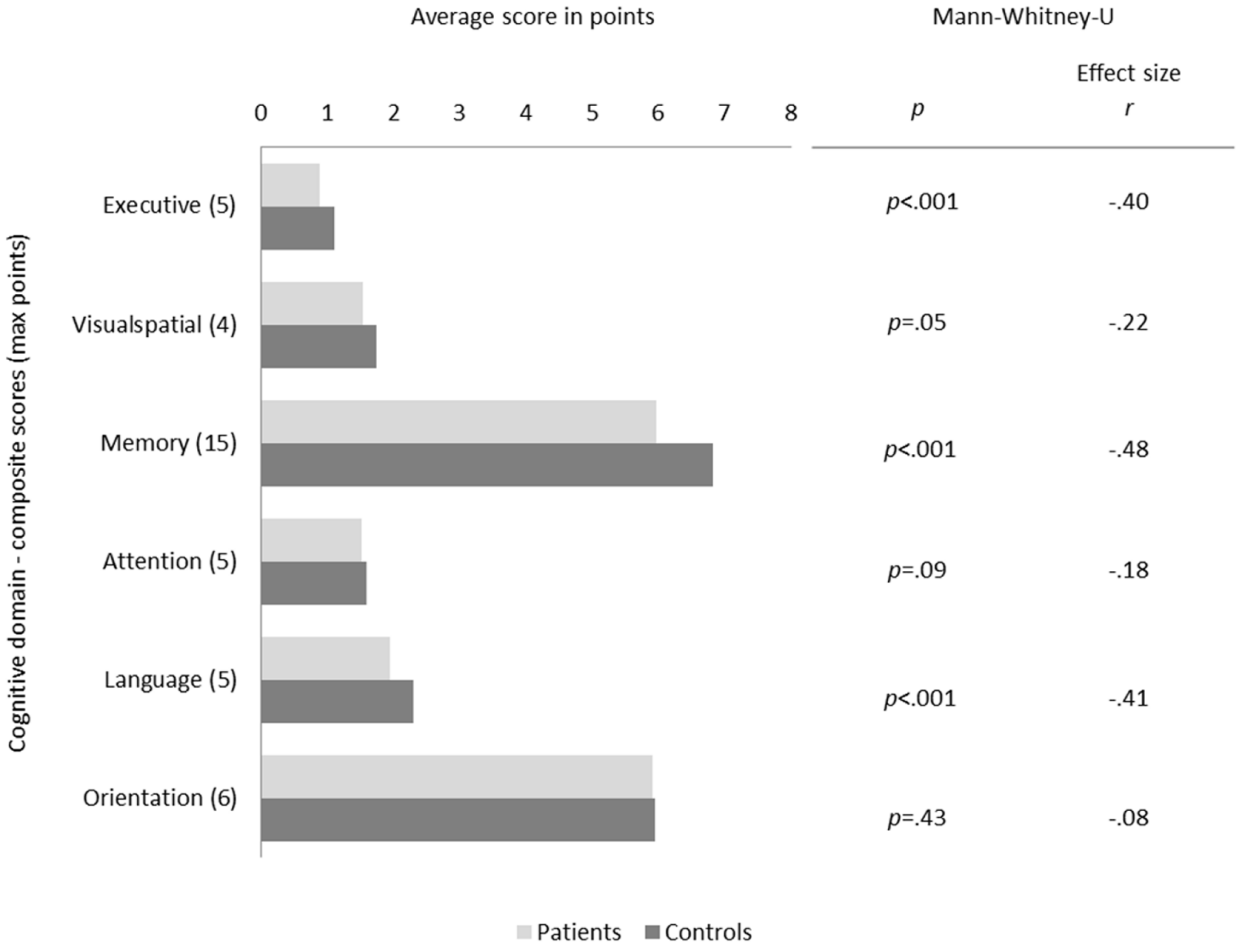
Group differences in cognitive domain composite scores of the Montreal Cognitive Assessment. The bar chart in figure 1 displays the average points scored by the patient and control group for each of the cognitive domains of the Montreal Cognitive Assessment (MoCA). The group differences were assessed using the non-parametric Mann-Whitney-U test with respective p-values showing a significantly (*p*≤.001) poorer performance of the patient group in the domains of executive functions, episodic memory and language. Notes. *Executive: Trail Making Test (TMT) B, verbal abstraction, digit span backwards, phonemic word fluency; Visuospatial: cube copying, clock drawing; Memory: immediate and delayed word recall; Attention: digit span forwards, letter cancelation, number subtraction; Language: sentence repetition, animal naming; Orientation: temporal and spatial orientation. Group differences were assessed using the Mann-Whitney-U test with a significance level of *p*≤.001.

HD patients achieved lower mean total scores in the MoCA than the control group. The deficits were especially prominent in the areas of executive functions, language ability and short-term memory capacity (Figure 1). The most difficult tasks appeared to be digit span backwards and forwards, phonemic word fluency, sentence repetition, verbal abstraction and immediate and delayed word recall (see Table S2 in File S1). No significant differences could be identified between groups in respect to visuospatial tasks, naming, level of attention (as tested in the letter cancellation and number subtraction tasks) or temporal and spatial orientation.

In the patient group there was a negative association between the MoCA total score and age (*r_s_* = −.38, *p*<.05), which was not found in the control group. There was a positive association (*r*_s_ = .28, *p*<.01) between the MoCA and education. From the association analysis between clinical variables and performance on the MoCA, only the CCI score for dialysis patients correlated negatively with the MoCA total scores (*r_s_* = −.53, *p*<.001), indicating that patients with a higher comorbidity score achieved lower results in the MoCA.

### Performance on the detailed neuropsychological test battery

In the detailed neuropsychological testing, patients performed worse than the control group in all cognitive domains (Table 2). Patient performance was worse in tasks such as immediate and delayed verbal and visual memory, semantic and phonemic word fluency, as well as performance on the TMT, reaction-timed alertness and the Stroop interference task. Cognitive dysfunction was identified in 29 patients (70%), whereby for 10 patients (24%) cognitive dysfunction could be classified as mild cognitive impairment and for 19 patients (46%) as severe cognitive impairment.

**Table 2:** Neuropsychological test results.

Neuropsychological domain/test	Test results				Mann-Whitney-U	
	N	Patients	N	Controls	*p*	Effect size *r*
Screening tests						
Montreal Cognitive Assessment (MoCA)	43	24.0 (4.0)	42	28.0 (3.0)	<.001*	−.573
Mini Mental State Examination (MMSE)	41	29.0 (2.0)	41	29.0 (2.0)	.03	−.247
Attention						
Test of attentional performance (TAP)						
TAP Phasic Alertness	35	276.0 (73.0)	39	243.0 (60.0)	.03	−.262
TAP Intrinsic Alertness	35	273.0 (51.0)	39	255.0 (64.0)	.01	−.301
Digit span forwards	41	7.0 (2.0)	42	8.0 (4.0)	.002	−.338
Verbal Memory						
California Verbal Learning Test (CVLT)						
CVLT Total learned	37	50.0 (16.0)	42	61.0 (15.0)	<.001*	−.415
CVLT Interference	37	5.0 (4.0)	42	6.0 (3.0)	.04	−.232
CVLT Immediate Recall	37	9.0 (6.0)	42	12.5 (6.0)	.002	−.327
CVLT Delayed Recall	37	11.0 (6.0)	42	12.5 (4.0)	.002	−.341
CVLT Recognition (correct)	37	16.0 (2.0)	42	16.0 (1.0)	.60	−.059
Non-verbal Memory						
Medical College of Georgia Complex Figures (MCGCF)						
MCGCF Immediate Recall	36	17.8 (12.4)	42	28.0 (13.5)	.004	−.325
MCGCF Delayed Recall	36	16.5 (12.6)	41	28.0 (13.5)	.008	−.266
Visuospatial						
MCGCF Copy	36	31.5 (8.5)	42	34.0 (2.0)	.02	−.264
Visual Object Space Perception (VOSP) Incomplete letters	40	20.0 (1.0)	42	20.0 (1.0)	.39	−.095
Language						
Boston Naming Test	39	15.0 (0.0)	42	15.0 (0.0)	.06	−.207
Executive						
Semantic word fluency	39	28.0 (17.0)	42	39.5 (12)	<.001*	−.409
Phonemic word fluency	39	14.0 (9.0)	42	21.0 (10.0)	<.001*	−.492
Digit span backwards	41	6.0 (2.0)	42	6.0 (1.0)	.25	−.126
Stroop Test						
Colour reading	33	35.0 (14.0)	41	32.0 (6.0)	.01	−.288
Colour naming	33	54.0 (17.0)	41	47.0 (12.0)	.01	−.285
Inteference	33	96.0 (49.0)	41	86.0 (28.0)	.02	−.269
Trail Making Test (TMT) Part A	39	51.0 (38.0)	42	32.5 (15.0)	<.001*	−.512
Trail Making Test (TMT) Part B	38	110.5 (96.0)	42	73.5 (37.0)	<.001*	−.427
Depression						
Hospital Anxiety and Depression Scale (HADS)						
Total score	42	11.0 (9.0)	42	4.5 (7.0)	<.001*	−.373
Anxiety	42	6.0 (5.0)	42	3.0 (5.0)	.02	−.266
Depression	42	4.5 (5.0)	42	1.0 (4.0)	<.001*	−.428
Fatigue						
Epworth Sleepiness Scale (ESS)	42	6.5 (5.0)	42	5.5 (5.0)	.05	−.217
Fatigue	21	5.0 (5.0)	34	2.0 (4.0)	.02	−.318

Notes. Test results presented as median (IQR).

*p-values with Bonferroni correction for multiple comparisons.

Significant difference at *p*<.001. Effect sizes, *r*: small (0.10–0.29), medium (0.30–0.49), large (>0.50).

Correlation analysis showed that the composite scores of the MoCA and the test battery correlated in the domains of executive functions (*r*_s_ = .60, *p*<.001) and memory (*r*_s_ = .53, *p*<.001), whereas the attention, language and visuospatial composites revealed no significant association. The MoCA and the MMSE total scores showed a moderate positive correlation (*r_s_* = .54, *p*<.001).

### Association between the MoCA and the detailed neuropsychological assessment

Linear regression analysis revealed that the composite score including executive functions tasks explained 39% (β = 0.62, R^2^ = .39, *p*<.001) of the variance in the MoCA and the non-executive functions composite score explained 34% (β = 0.58, R^2^ = .34, *p*<.001). Together, executive and non-executive functions accounted significantly for overall performance on the MoCA (β = 0.42, R^2^ = .44, *p*<.001), while the covariates age (β = 0.26, R^2^ = .07, *p*<.05) and education (β = 0.23, R^2^ = .05, *p*<.05) only marginally affected variance in performance. Sleepiness and fatigue did not show a significant effect on cognitive performance, yet high scores on the depression scale had a mild effect (β = −0.36, R^2^ = .13, *p*<.001).

### ROC analysis and predictive values

**Figure 2:**
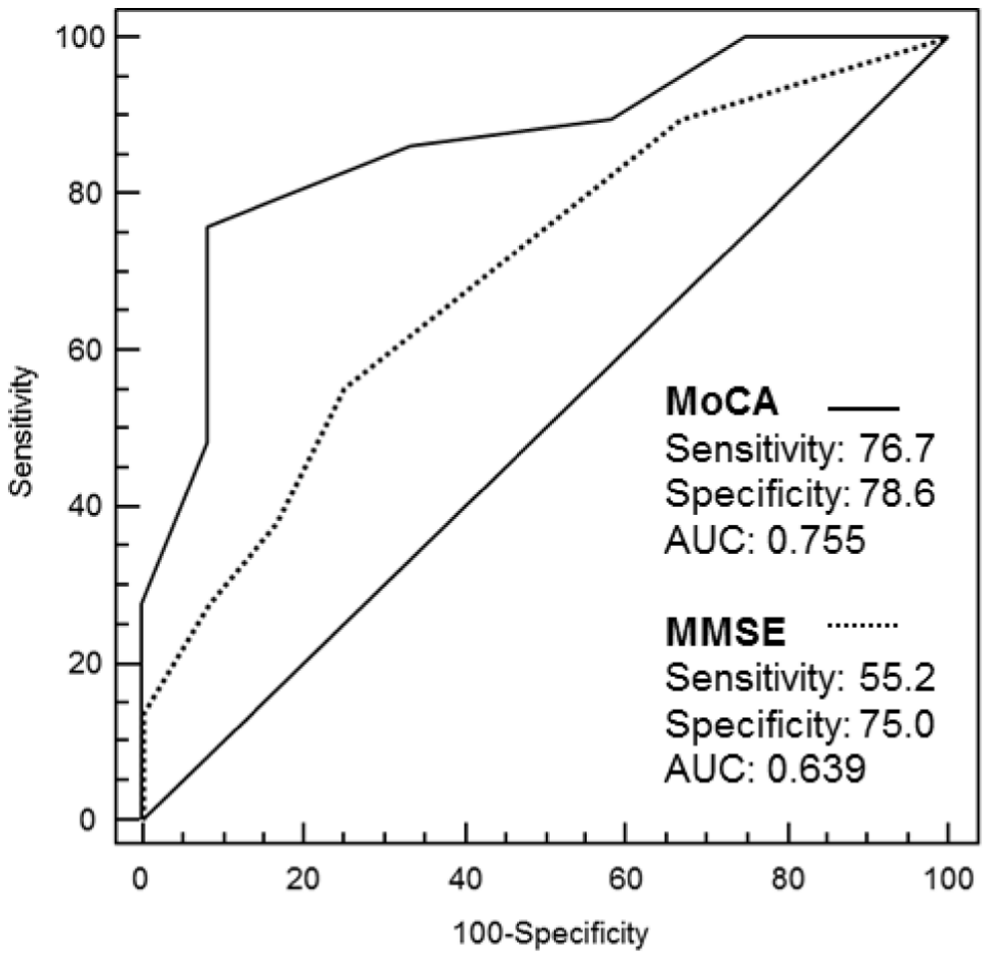
ROC curves for the cognitive screening tests Montreal Cognitive Assessment and Mini-Mental State Examination. The receiver operating characteristics curves for the Montreal Cognitive Assessment (MoCA) and the Mini-Mental State Examination (MMSE) illustrate the discriminative capacity of each of the screening tests, displaying their individual sensitivity, specificity and area under the curve (AUC). The MoCA shows good levels of sensitivity and specificity, as well as an overall greater AUC than the MMSE, while the MMSE presents a high specificity and relatively low sensitivity. Notes. MoCA = Montreal Cognitive Assessment; MMSE = Mini-Mental State Examination; AUC = Area under the curve.

The ROC analysis disclosed an optimal cut-off for the MoCA at ≤24 points (Table 3), with a sensitivity of 76.7%, specificity of 78.6% and an area under the curve (AUC) of 0.755 (95% CI, 0.602–0.872). In comparison, the MMSE only achieved a sensitivity of 55.2%, and a specificity of 75.0%, and AUC of 0.701 (95% CI, 0.538 to 0.834) for an optimal cut-off of ≤28 points (Figure 2). Using a cut-off value of ≤24 for MoCA results, a total of 26 patients (59%) scored 24 points or lower, while 18 patients achieved a higher score. Respectively, a PPV of 0.88 and NPV of 0.61 could be calculated for the patient cohort. We identified 19 patients (46.3%) with a MMSE score of ≤28 points.

**Table 3:** Criterion (cut-off) values and coordinates of the ROC curves of MoCA and MMSE.

Screening test	Criterion	Sensitivity	95% CI	Specificity	95% CI
MoCA	≤22	46.67	28.3–65.7	78.57	49.2–95.3
	≤24*	76.67	57.7–90.1	78.57	49.2–95.3
	≤25	86.67	69.3–96.2	57.14	28.9–82.3
	≤26	90.00	73.5–97.9	35.71	12.8–64.9
MMSE	≤27	37.93	20.7–57.7	83.33	51.6–97.9
	≤28*	55.17	35.7–73.6	75.00	42.8–94.5
	≤29	89.66	72.6–97.8	33.33	9.9–65.1

Note:

*optimal cut-off score based on maximal sensitivity and specificity using the receiver operating characteristic (ROC) analysis.

## Discussion

Here we demonstrate that the MoCA is a valid and well-suited screening tool for cognitive impairment in HD patients. The MoCA was capable of discriminating between HD patients with and without cognitive impairment, as defined by performance on a comprehensive neuropsychological test battery, presenting good sensitivity and specificity levels, as well as a good concurrent validity. In contrast, the MMSE revealed only a weak group discriminative power.

Our main results confirm that the MoCA is able to reliably identify cognitive impairment in CKD patients undergoing hemodialysis. We could establish an optimal cut-off of ≤24 points out of a 30 points maximum, which is lower than the cut-off value of ≤26 described in the original data collected in a population of patients with AD and mild cognitive impairment (MCI) [11]. Given that cut-off values are population specific [39], several other studies have determined lower values in different populations, e.g. a cut-off of 23.5 in a population with MCI [19] and of 21/22 in a population with cerebral small vessel disease [17]. With a good sensitivity (76.67) and specificity (78.57) our findings are consistent with previous research, where the MoCA's sensitivity in detecting cognitive impairment ranged from 56% to 100%, while specificity varied between 29% and 87%, depending on the study population [11], [18], [40]. More specifically, in the detection of vascular cognitive impairment the MoCA presented a specificity of 68% and lower sensitivity of 56% in a population with silent cerebral infarction [40].

The detailed cognitive assessment showed a distinct difference in achievement between groups. This tendency was equally present in the MoCA results, whereas performance did not differ between groups for the MMSE. The prevalence of cognitive impairment of 70% in this cohort, as classified by the testing battery, corresponds to the levels of cognitive dysfunction stated in previous studies with larger cohorts of dialysis patients [5], [7]. The results presented by the MMSE equally match previous characterizations of CKD patient cohorts, where the level of cognitive dysfunction was measured at 30% when only using the MMSE as a diagnostic measure [4], [6]. The prevalence of cognitive dysfunction in this patient cohort appears, therefore, to be similar to previous findings and allows the assumption that it is representative for this population. Correlation analysis showed a strong relationship between MoCA results and the detailed neuropsychological testing, especially for memory and executive functions, which may suggest good diagnostic ability in these areas. There remains, nevertheless, a limitation for interpretation of correlation between tests, due to a partial overlap between tasks that may affect association analysis.

The profile of cognitive deficits found in our patient population, including significant impairment in executive functions, processing speed, word fluency and short-term verbal and non-verbal memory capacity, stands in accordance to previous findings [3], [41] and similar results have been associated with vascular disease as a potential cause of cognitive impairment in HD patients [15], [42]. Given the high prevalence of executive dysfunction in CKD patients and our findings that performance in the MoCA was predominantly dependent on executive ability (as has been described before [43]), we believe that this is one of the strengths of the MoCA in comparison to other generally used cognitive screening instruments. In this regard, the focus of the MoCA on executive functions is believed to be a reason why it is far superior to the MMSE in particular clinical populations, given that the MMSE lacks the assessment of such cognitive domains and has a tendency to produce ceiling effects [43], [44].

This may be particularly important considering that the majority of previous studies with CKD patients have included the MMSE as the preferred cognitive screening test [42] or even the only cognitive instrument [45], [46]. Methodologically, the fact that the MoCA and the MMSE subtests partially overlap may limit considerations of their differential discriminant validity, but it should be noted that the original rationale behind MoCA, through the inclusion of a wider range of cognitive domains than the MMSE, especially considering the executive functions, already entails some advantage in terms of sensitivity and specificity.

Based on our current results, and as others have suggested [8], the MMSE may be inadequate for this population and may underestimate the extent of cognitive impairment. Nevertheless, a recent study showed that the MMSE was able to detect progression of cognitive impairment in HD patients [47], but may be less suited for early detection of mild cognitive deficits in other populations [48]. In a similar fashion, another screening test, the Kidney Disease Quality of Life Cognitive Function (KDQOL-CF), was found to be inadequate for assessing cognitive function in HD patients, due particularly to the lack of executive tasks [49].

A procedural advantage of the MoCA over the MMSE may also be the availability of alternative versions [25], which enables longitudinal testing while avoiding practice effects. Longitudinal testing is especially interesting in HD patients, as fluctuations in cognitive performance during the hemodialysis cycle have been identified [20], [50].

Concerning predictive clinical variables, we were able to show that high comorbidity rates were associated with lower performance on the MoCA, which opens the question of the etiological or mediator role that different comorbidities play in cognitive impairment in such a heterogeneous group.

Other interesting findings were that although levels of fatigue and sleepiness were higher in the patient group, which is in line with statements from earlier studies that fatigue is a common problem in HD patients [3], [51], we did not find fatigue or sleepiness to influence cognitive performance. It is possible that conducting the assessment on a dialysis-free day avoided post-dialytic fatigue and enabled a more objective assessment of cognitive performance. In contrast, a significant association was found between lower performance and higher levels of depressive symptoms, which supports assumptions that depression may be a relevant co-factor for poor cognitive performance and should also be screened for in CKD patients [52]–[54]. This is especially important when taking into account the prevalence of depression of up to 25% in HD patients [51] and the number of associated problems, including lower quality of life and higher hospitalization and mortality rates [54].

Further strengths of our study include the detailed neuropsychological testing, which enabled a comprehensive evaluation of cognition and the interpretation of concurrent validity, as well as the inclusion of a healthy control group that served as reference for cognitive performance. The MoCA has recently been suggested for cognitive screening in a small cohort of HD patients showing similar results for MoCA with an average of 24 points before dialysis [20], yet the emphasis was put on testing environment and variation in performance during the HD cycle and only the MMSE was used as a reference. This is therefore a valuable addition, as there is no precedent data on the psychometric characteristics of MoCA in comparison to detailed cognitive analysis and clinical characterization of a moderate size sample of HD patients. Limitations are, nevertheless, the relatively small sample size, which makes it difficult to generalize our findings. Although our results allow the assumption of cognitive impairment in HD patients below the cut-off value of 24 points in the MoCA, a clear distinction between MCI and dementia cannot be made in this study, due to the exclusion of patients with dementia. Equally, one must mention again the concordance of tests used in the screening tests and the detailed test battery that may influence correlation analyses in form of a positive bias. Our decision to enter education as a covariate rather than using the education correction for MoCA values, for which there is no normative data for a German population, may be seen as inadequate as there is a slight difference in educational levels between groups. Furthermore, data on subjective cognitive impairment, and clinical data were limited, the latter especially for the control sample, reducing our ability to assess variables, such as cardiovascular risk factors and hemodialysis parameters, in more detail. Further research is warranted to confirm these findings in a larger patient sample, possibly including non-dialysis CKD patients to further define indications for cognitive screening with the MoCA.

Cognitive impairment is a highly relevant clinical factor for disease progression in HD patients, possibly also affecting daily-life activities, thereby impeding adherence to therapeutic regimes and compromising quality of life. With the aims of improvement of individual outcome and optimizing the utilization of medical care resources, a brief cognitive screening test for HD patients is an essential addition to clinical practice. We presume that the MoCA, granted its validity and psychometric characteristics, is very suitable for this purpose, as it represents a multi-dimensional screening test that not only includes relevant tasks for the assessment of the HD population, but also offers the possibility of longitudinal measurements with available alternate versions.

In conclusion, the MoCA is an adequate screening tool for a brief bedside evaluation of global cognitive performance in HD patients and has the potential to assist in the daily clinical care of HD patients.

## Supporting Information

File S1 **Supplemental files. List S1.** Order of test administration. **Table S1.** Description of neuropsychological test battery. **Table S2.** Test results of MoCA subtests.
(DOCX)
