# Supplementary material for: The Montreal Cognitive Assessment (MoCA) - A Sensitive Screening Instrument for Detecting Cognitive Impairment in Chronic Hemodialysis Patients
Source: PLoS One. 2014 Oct 27;9(10):e106700. doi: 10.1371/journal.pone.0106700 (PMC4209968; doi:10.1371/journal.pone.0106700)
Supplement: File S1 — Supplemental files. List S1. Order of test administration. Table S1. Description of neuropsychological test battery. Table S2. Test results of MoCA subtests. (DOCX) [file pone.0106700.s001.docx]

**Supplement**

**Table S1. Description of neuropsychological test battery**

| **Neuropsychological test** | **Brief description** |
| --- | --- |
| **Montreal Cognitive Assessment (MoCA)** | Ten-minute test including trail making B, cube copying, clock drawing, animal picture naming, digit span backwards and forwards, serial subtraction, selective attention, sentence repetition, phonemic word fluency, verbal abstraction, 5-word learning and delayed recall task and questions to spatial and temporal orientation. |
| **Mini-Mental State Examination (MMSE)** | Screening test containing questions to spatial and temporal orientation, serial subtraction, immediate word recall, object naming, figure copying and oral command comprehension. |
| **Test of attentional performance (TAP)** | Computer based reaction time measurement |
| **California Verbal Learning Test (CVLT)** | Repetition and retention of a word list containing 4 categories of words. Cued and delayed recall after 3 and 30 minutes and a word recognition task. |
| **Medical College of Georgia Complex Figures (MCGCF)** | Copy and immediate and delayed recall of a complex geometrical figure. |
| **Visual Object Space Perception (VOSP) Incomplete letters** | Identification of visually degraded stimuli. |
| **Boston Naming Test** | Naming of ink drawings. |
| **Semantic word fluency** | Spontaneous production of words of a given category in two minutes. |
| **Phonemic word fluency** | Spontaneous production of words with a given first letter in two minutes. |
| **Digit span forwards** | Number sequence repetition. |
| **Digit span backwards** | Repetition of a number sequence in reverse order. |
| **Stroop Test** | Completion time of color word reading, naming and naming of mismatched pairs of word and color. |
| **Trail Making Test (TMT) – Part A** | Connection of 25 consecutive numbers in the correct order. |
| **Trail Making Test (TMT) – Part B** | Connection of 25 alternating numbers and letters. |
| **Hospital Anxiety and Depression Scale (HADS)** | Questionnaire on depression and anxiety symptoms. |
| **Epworth Sleepiness Scale (ESS)** | Questionnaire on day-time sleepiness. |
| **Fatigue scale** | Subjective fatigue level on a scale of 0-10. |

**Table S2. Test results of MoCA subtests**

|  | Patients | | | | Controls | | | Mann-Whitney-U |
| --- | --- | --- | --- | --- | --- | --- | --- | --- |
| Subtests | Median | | IQR | | Median | IQR | | *p* |
| MoCA total score | 24.0 | 4 | | 28 | | | 3 | ≤.001* |
| MoCA trail making test B | 1 | 0 | | 0.88 | | | 0 | .419 |
| MoCA cube copying | 1 | 1 | | 1 | | | 0 | .141 |
| MoCA clock drawing | 2.5 | 1 | | 3 | | | 1 | .095 |
| MoCA naming | 3 | 0 | | 3 | | | 0 | .150 |
| MoCA immediate word recall | 9.5 | 1 | | 10 | | | 0 | .005* |
| MoCA digit span forwards | 1 | 0 | | 1 | | | 0 | .047* |
| MoCA digit span backwards | 1 | 0 | | 1 | | | 0 | .002* |
| MoCA cancelation | 1 | 0 | | 1 | | | 0 | .179 |
| MoCA number subtraction | 3 | 0 | | 3 | | | 0 | .505 |
| MoCA sentence repetition | 1 | 1 | | 2 | | | 1 | ≤.001* |
| MoCA phonemic word fluency | 0 | 1 | | 1 | | | 0 | ≤.001* |
| MoCA abstraction | 2 | 1 | | 2 | | | 0 | .040* |
| MoCA delayed recall | 3 | 4 | | 4 | | | 2 | .002* |
| MoCA cued recall | 4 | 3 | | 5 | | | 1 | .006* |
| MoCA recognition | 5 | 1 | | 5 | | | 0 | .055 |
| MoCA orientation | 6 | 0 | | 6 | | | 0 | .434 |

Notes. IQR= interquartile range, **p-*values significant at *p*≤.05

**List S1. Order of test administration**

1. Montreal Cognitive Assessment (MoCA)

2. Test of attentional performance (TAP)

3. California Verbal Learning Test (CVLT) – Total learned, Interference, Immediate recall

4. Medical College of Georgia Complex Figures (MCGCF) – Copy

5. Semantic word fluency

6. MCGCF – Immediate recall

7. Phonemic word fluency

8. Trail Making Test (TMT) – Part A

9. Trail Making Test (TMT) – Part B

10. Digit span forwards

11. Digit span backwards

12. Boston Naming Test

13. Visual Object Space Perception (VOSP) Incomplete letters

14. Stroop Test

15. CVLT – Delayed recall and Recognition

16. MCGCF – Delayed recall

17. Mini-Mental State Examination (MMSE)

18. Hospital Anxiety and Depression Scale (HADS)

19. Epworth Sleepiness Scale (ESS)
